# Bibliographical Notices

**Published:** 1846-04

**Authors:** 


### BIBLIOGRAPHICAL NOTICES.

*Homœopathy, Allopathy, and Young Physic.* By John Forbes, M.D., F.R.S.; one of the Editors of the Cyclopædia of Practical Medicine; Editor of the British and Foreign Medical Review, &c. &c. 12mo. pp. 121.

In our last number we noticed the 'Medical Problems' of the Messrs. Griffin, and stated that we were glad to see such productions, leading us, as they do, to investigate principles; and often to mistrust isolated or agglomerated facts, which, after all, have been the bane of medical science. We mean, of course, so-called "facts;" and who is to discriminate between the true and the false? Each recorder considers himself qualified to observe, and would feel insulted if told, that his observations are not as trustworthy as those of any other individual. Who, then, is to sift, so as to enable us to embrace the authentic, and discard the doubtful? It would be presumptuous in any one to attempt it; and hence a 'registrar-general,' like M. Valleix, may pretend to analyze, and oracularly promulge his analyses; and after all may merely congregate the records—many of them most fallacious—of all. Homœopathy—if we credit its sectaries—is founded on facts. It is true it has a series of speculations—almost all of which are eminently ridiculous—to connect those facts together, so as to give it the semblance of a science; yet it is no less true, that it appeals in all cases to positive observed results; and every convert to it professes to have been changed in his belief by witnessing undoubted 'facts'—cures that have been accomplished by its agency; for few would be hardy enough to admit that their minds had been led captive by its 'seductive' theories. And shall we reject all these homœpathic 'facts,' because they are brought forward by men who profess another faith, but affirm, that they observed honestly, and registered as honestly, what they witnessed? What greater right—it may fairly be asked—have we to discard their statistical details than they have to reject ours? Both parties, it ought to be presumed, are honest; whilst the homœopathists ought—we think—to permit a greater latitude of doubt to be entertained in regard to their statements, in consequence of the absurd doctrines with which they are associated. Homœopathy, however, is not the only curative system that appeals to facts. There never has been brought forward a solitary nostrum—howsoever unworthy it may have really been—that has not had multitudes of 'facts,'—or what have been called 'facts'—to support it. Disease has disappeared under its use, as under the agents administered by the homœopathist. It has vanished also under the remedies prescribed by the regular physician; yet experiments have rarely been made to determine how far the results were really dependent upon the articles administered; and how much upon the action of that recuperative power—that "divinity which stirs within us"—and without which all therapeutical agents would be useless, and often worse than useless.

The object of the pamphlet before us is to direct our thoughts into this channel. It appeared originally in the British and Foreign Medical Review, for January, 1846, of which its author is the able editor; was published in England in pamphlet form, and has been reprinted in this country. We are glad of it; for, doubtless, by interested parties it would have been misunderstood, as it has been, or be garbled, or both. Now, it is put at such a price that all may obtain it, and read the whole article for themselves; and we will venture to say, that no unprejudiced person can rise from its perusal without being satisfied, that its author is a learned, accomplished, and scientific physician; full of love for the profession of his choice; ardent in the furtherance of every thing that may tend to its improvement; but, above all things, solicitous to discover the truth, and to be instrumental in its promulgation.

The great aim of the pamphlet is to show, that a reform is needed, not in medical polity—not the reform question which has long agitated, and still agitates the profession, both here and elsewhere—but in medical practice; and that the heroic therapeutics adopted more or less everywhere should be abandoned, and more confidence be placed in great principles of hygiene and therapeutics, and especially in the natural or instinctive powers, which, after all, are our anchors of hope in the management of disease, and are always more trusted to by the experienced practitioner than by one who is just commencing his career. As in politics the young are apt to be bustling, energetic reformers; the older quiet, confiding and conservative; so, in medicine, polypharmacy and 'action, action, action,' are with the young practitioner; diminished confidence in the adaptation of special remedial agents for special morbid conditions, and more confidence in the instinctive powers of the organism, with the older and more experienced.

In referring to a few of the more open sources of evidence of the most unequivocal kind, that nature *can* cure disease, the author remarks:

> "Lastly, we must advert to what is, perhaps, the most extensive and valuable source of all—the actual practice of the more scientific physicians of all ages, in the latter part of their career—men of philosophic minds as well as of much experience. It is well known, from the history of physic, that a large proportion of men of this class have, in their old age, abandoned much of the energetic and perturbing medication of their early practice, and trusted greatly to the remedial powers of nature. The saying of a highly respected and very learned physician of Edinburgh, still living at an advanced age, very happily illustrates this point. On some one boasting before him of the marvellous cures wrought by the small doses of the Homœopathists, he said, 'this was no peculiar cause for boasting, as he himself had, for the last two years, been curing his patients with even less, viz.: with nothing at all.' " p. 90.

After a brief sketch of Hahnemann, the founder of the Homœopathic System; and an inquiry into the evidence—doctrinal and practical—afforded by its believers, Dr. Forbes concludes "that the curative powers of nature suffice to explain all the triumphs of homœopathy." To this, we apprehend, every reflecting and unbiassed mind, that has inquired at all into the subject, must have already arrived. Over and over again our own views on this subject have been recorded in fugitive essays, as well as in more elaborate productions. As regards the folly of the doctrines and infinitesimal remedial agencies of the homœopathists, the author is as fully impressed as their most bitter acknowledged opponents, but he accords, we think, to Hahnemann more merit as a reformer than he deserves. To him he considers the profession to be mainly indebted for the abandonment of perturbating methods of treatment previously in constant use. On this side the Atlantic the reform was certainly not introduced by Hahnemann. It was commenced or promoted by the more general adoption of the views of Broussais, who entered the list in uncompromising hostility with the perturbators, and especially with those whose disturbing agents were chiefly directed to the lining membrane of the alimentary canal; but its decadency was mainly owing to a better system of observation and reflection, and to influential teachers promulgating *ex cathedrâ* the importance of considering the physician to be the interpreter and minister of nature, and not the disturber of her efforts.

Perhaps, too, the author has assigned undue importance to the trifling amount of evidence adduced in favour of homœopathic practice by Drs. Fleischmann and Henderson—both homœopathists. Admitting their statistics, however, to be true, his conclusions follow irresistibly: and who shall say that they are not to be credited?

As the pamphlet, or the Review whence it is extracted, will probably be read by most of our readers, we shall not occupy further space with its consideration. We would, however, direct especial attention to the concluding portion—the "*cogitanda*, *excogitanda*, and *agenda*"—twenty in number, with every one of which we are disposed to accord. We recommend all the observations to be perused and reperused. They emanate from one of no little standing, professionally and socially, and are unquestionably the honest convictions of their author. "We have said only"—he observes—"what we believe to be true; and if what we believe is in reality the truth, the promulgation of it cannot lead to evil. Truth is good. If the art of medicine, as we profess and practise it, cannot bear investigation, and shrinks before the light of truth, from whatsoever quarter it may come, it is high time that it should cease to be sanctioned and upheld by philosophers and honest men. If, on the contrary, it be true and good—even if it be but partially true and moderately good—the stirring torch of inquiry and the stimulus of opposition cannot fail to benefit it in the end." p. 78. *

*Julii Vogel Icones Histologiæ Pathologicæ. Tabulæ Histologiam Pathologicam illustrantes, &c. &c. Eslaüterungstafeln zur pathologischen Histologie mit vorzüglicher Rücksicht auf sein Handbuch der pathologischen Anatomie;* herausgegeben von Dr. Julius Vogel, ausserordentl. Professor der Medizin in Göttingen, &c. 4to. S. 128. Leipzig, 1843.

*Physiologie pathologique ou Recherches cliniques, expérimentales et microscopiques sur l'Inflammation, la Tuberculisation, les Tumeurs, la formation du Cal, &c.* Par A. Lebert, &c. Accompagné d'un Atlas de vingt-deux planches gravées. 2 vol. pp. 544, 515. Paris, 1845.

The works before us—one from a German, the other from a French observer—are illustrative of the tendencies of the day, to apply the microscope to the elucidation of the intimate organization, both in health and disease. The very term "pathological histology" was unknown a few years ago. The most striking changes in the organs were then recorded by the pathological anatomist; at present, he proceeds more minutely, and endeavours to depict the intimate alterations of tissue that occur in disease, and can only be accurately appreciated by the employment of the microscope. We have before stated, that to this instrument and to organic chemistry, we may look with encouraging anticipations for an improvement of our knowledge of the diseased condition. Already they have thrown much light on healthy histogeny.

Dr. Vogel's plates are twenty-six in number. They are well executed copper plate engravings, containing, in the whole, two hundred and ninety-one figures, of which two hundred and seventy are delineated after nature. Of the plates, twelve are illustrative of general, and the remainder of special pathological histology.

The work of M. Lebert is much more voluminous. It is devoted to the same objects as that of Vogel; but enters, in the text, into a more extended investigation of the various subjects of which it treats. The sections on tuberculosis are especially full. The various degenerations of tissues—and particularly tumours—are likewise described in ample detail; and to the whole are appended memoirs on the formation of callus; on tinea; hydatids of the liver, and on the elementary globules, as observed in pathological products.

The plates in the Atlas are well executed. They are on copper, and twenty-two in number.

---

*A Manual of Chemistry.* By Richard D. Hoblyn, A. M., oxon., author of a "Dictionary of terms used in Medicine and the Collateral Sciences." 12mo., pp. 235. Samuel S. and William Wood. New York, 1846.

Of all the sciences, Chemistry, perhaps, lays claim to the most general utility. Important as a knowledge of it undoubtedly is to the physician, it is scarcely less so to the farmer, the artist and the artizan. Whatever, therefore, is calculated to facilitate the acquisition of a knowledge of so important a branch of human learning, is entitled to be regarded with particular favour. We are no advocate of superficial knowledge,—of that smattering which too many seem to regard as perfection,—but contend with the sturdiest for "drinking deep," more especially in what concerns that which constitutes an individual's daily occupation. Still, in studying any branch of science, it is convenient to begin with a general survey of the subject, before entering upon the minute investigation of particular parts; just as the artist traces the outlines of his picture before employing the various colours which give to the several parts their distinctive features. In preparing a book on this plan, there is always much difficulty in determining the extent to which descriptions and discussions should go—what to include, and what to omit. Elaborate detail discourages the mind of a beginner by the labour it promises, whilst undue brevity fails to interest it, by the omission of that which is necessary to render the subject clear and intelligible. Hence, a treatise on any art or science, to be adapted to the wants of the beginner, should contain just enough of detail, in facts and illustrations, to convey a correct knowledge of its *principles*; referring the investigator to more elaborate works for the materials necessary for a profound acquaintance.

It is rarely that we meet with a manual so well adapted for the object of its publication, as the work the title page of which we have given above. It has been prepared, we are told by the author, "for the purpose of affording public or private instruction in the principles of chemistry. The most important facts and theories of the science are stated, in as condensed a form as the subject admits;" and a series of recapitulatory questions is appended to each chapter, for the examination of students.

The work is divided into *three* sections; and beside a short preface and table of contents at the beginning, we have a glossary and index at the end of the volume.

*Section* 1*st*. Treats of the imponderable agents—heat, light, and electricity.

*Section* 2*d*. Chemistry of Inorganic Substances—first, of the non-metallic substances, as OXYGEN, HYDROGEN, NITROGEN, CARBON, Sulphur, *Phosporus*, etc. etc.; secondly, of the *Metallic elements*, as Potassium, Iodine, Lithium, Calcium, etc. etc.

*Section* 3*d*. On Chemistry of Organic Substances—first, of Vegetable Chemistry; and secondly, of Animal Chemistry.

The work is tersely written, and with so much conciseness, that a great amount of matter is comprised in comparatively a few pages. We know, indeed, of no work on the same subject, that contains so clear and comprehensive an account of the principles of chemistry, within any thing like so brief a space.

*Elements of Materia Medica and Therapeutics.* By Edward Ballard, M. D., etc. etc., and Alfred Baring Garrod, M. D., etc. etc. *With Additions and Alterations.* By R. Eglesfeld Griffith, M. D. 8vo. pp. 516. Philadelphia. Hogan & Thompson. 1846.

This is an American edition of a work just out from the London press, and designed for the especial use of students. The object of the authors in its preparation has been to present "the essential points of instruction," conveyed in fewer words than in extended treatises, and less encumbered with extrinsic matter. "The writers desire that it should be looked upon as strictly elementary; and, in so far as the description of the drugs is concerned, nothing more than a compilation." As an "*outline*," as the authors justly style it, the present volume is in the highest degree creditable. The account of the various drugs, as far as it goes, is generally correct, and the chemical and pharmaceutical details remarkably accurate. The only question in the matter is, whether a mere outline is sufficient for the purposes of the student or the practitioner, either for study or reference? We think not. We are of the opinion of Dr. Johnson, that a book should "contain all the reader wishes to be informed of upon the subject laid before him." Meagre details never satisfy the mind, particularly on subjects that demand close investigation and an intimate acquaintance to enable a man to discharge the responsible duties of his daily avocation. For students of medicine especially, it seems to us, that a work designed for their use should be full and complete, as to everything that is *essential* for them to know, and where principles are concerned, some discussion is necessary to enforce them upon the mind. This class of readers, too, rarely have the means to purchase, or the time to examine, many works on any particular subject, and whatever merit there may be in brevity, when carried to an extent that excludes important facts and necessary illustrations, the youthful mind is embarrassed rather than instructed. Notwithstanding these objections to the work before us, it is but justice to say, that it contains much accurate and useful information, and to one thoroughly acquainted with the subject, who requires little more than brief notes to refresh his memory, will be found of essential service.

To adapt the work more fully to the use of American students, the processes and preparations directed in the United States Pharmacopœia have been added to those of the London College, by the editor, Dr. Griffith. A number of medicinal substances, of native origin, have also been inserted in their proper places, and the descriptions of most others greatly extended or modified. While we are not prepared to justify this liberty with the text by any but the authors themselves, we freely admit that the additions and alterations, made by the editor, add much to the value of the work. The mechanical execution of the present edition is very creditable to the publishers. White paper and good clear type are great recommendations, especially to those who are compelled to read at night, even with the advantages of gas-light.

